# The relevance of basic psychological needs and subject interest as explanatory variables for student dropout in higher education — a German case study using the example of a cooperative education program

**DOI:** 10.1007/s10212-022-00671-4

**Published:** 2023-01-04

**Authors:** Steffen Wild, Sebastian Rahn, Thomas Meyer

**Affiliations:** 1 Research Unit of Psychology and Diagnostics, Department of Rehabilitation Sciences, TU Dortmund University, Emil-Figge-Str. 50, 44227 Dortmund, Germany; 2 Faculty of Social Work, Baden-Wuerttemberg Cooperative State University Stuttgart, Stuttgart, Germany

**Keywords:** Self-determination theory, Subject interest, Dropout, Basic psychological needs, Person-object theory of interest

## Abstract

Student dropout in higher education is a challenge for higher education systems. In recent years, there has been an increasing focus on analyzing motivational aspects in order to counteract dropout. However, the detailed impact mechanisms and processes of motivation on dropout have not been sufficiently researched. For example, there is very little research analyzing the preconditions of motivation and their influence on motivation as well as their eventual influence on dropout. From the background of self-determination theory and the person-object theory of interest, this study analyzes the effects of satisfying the three basic psychological needs on dropout via subject interest. We use data from a cross-sectional design with *N* = 2662 cooperative students in their first academic year. Our analysis identifies a direct effect of relatedness and subject interest on dropout. Furthermore, indirect effects of satisfying basic psychological needs, specifically, autonomy and relatedness, on dropout via subject interest are noted. We evaluate our results in the context of the current state of research and discuss implications.

Student dropout in higher education is a heavily discussed topic, and no convincing measure has yet been found to effectively counteract it (Kehm et al., 2019; Neugebauer et al., 2019; Seidman, 2012). International research on student dropout still indicates high rates of approximately 30% dropouts, like “around 70% of students who enter a tertiary program graduate with a first degree at this level” (OECD, 2013, p. 64). Therefore, “25–45% of incoming college freshmen never graduate” (Nes et al., 2009, p. 1887), and the latest research aligns with these rates, with approximately 30% dropout in Germany across a wide range of study programs (Heublein et al., 2020). Research has also shown that the highest risk for student dropout is in the first academic year (Chen, 2012; Willcoxson et al., 2011; Wild & Schulze Heuling, 2020). Moreover, scientists highlight that dropout can be seen as a process with multiple causes (Heublein, 2014; Mashburn, 2000). One current suggestion regarding a motivational decision process is due to Bäulke et al. (2021); based on empirical evidence, they posit five phases, which they label (a) non-fit perception, (b) thoughts of quitting, (c) deliberation, (d) information search, and (e) final decision.

Motivation influences a person’s educational career, including their academic performance (Richardson et al., 2012; Schneider & Preckel, 2017) or participation in educational programs (Harackiewicz et al., 2002), but it decreases during the first academic year (Benden & Lauermann, 2021; Robinson et al., 2019). In recent years, motivation is seen more and more as an important factor for student dropout (Benden & Lauermann, 2021; Dresel & Grassinger, 2013; Schnettler et al., 2020). Along with this, Xu et al. (2021) postulate a growth in research focusing on student motivation in higher education and on the question of what works to motivate students for a long time. Schiefele et al. (2018) approach the question by modeling of motivational processes. However, Klein and Stocké (2016) note that dropout intentions, rather than actual dropouts, are used in such research.

Our research addresses these issues and attempts to close the implicit gaps. Based on self-determination theory (SDT; Ryan & Deci, 2000), we predict that the satisfaction of students’ basic psychological needs (BPNs) could be a relevant factor in influencing subject interest development, in accordance with the person-object theory of interest (POI; Krapp, 2002), which, in turn, affects student dropout, based further on situated expectancy-value theory (Eccles & Wigfield, 2020). In other words, subject interest is expected to mediate between the satisfaction of BPNs and the likelihood that a student will dropout. In this study, we test our hypotheses in subpopulations, in this case, cooperative students, for robustness. In addition, we work out the first indications for a process of motivation development. Furthermore, we work with actual dropouts and focus on the first academic year. At least, we believe this contributes to the analysis of potential remedies for high dropout rates.

## Theoretical framework and empirical results

### Basic needs theory and dropout

In the developed of SDT by Ryan and Deci (2017a), the framework of the three BPNs is one part of six mini-theories (Sheldon & Prentice, 2019). Cognitive evaluation, organismic integration, causality orientation, goal contents, and relationship motivation are the other five theoretical approaches. This concept postulates that when the three BPNs, autonomy, competence, and relatedness, are satisfied, motivational aspects will increase as well as social values, vitality, and cognitive performance (Ryan & Deci, 2017b). According to Deci et al. (1991), competence can be succinctly described as understanding how to attain various external and internal outcomes and being efficacious in performing the required actions. Relatedness means developing secure and satisfying relationships with other people. Finally, autonomy is defined as self-initiating and self-regulating of one’s own activities.

Empirical research supports the effect of BPNs on dropout. Numerous studies have been done in the field of education. In research focusing on school, work, and parental support, it has been shown that BPNs influence school dropout (Ricard & Pelletier, 2016; Taylor et al., 2012). Other studies in the context of school research have highlighted the influence of autonomy and competence on dropout (Alivernini & Lucidi, 2011; Gueta & Berkovich, 2022; Hang et al., 2017).

Higher education research on the association between BPNs and student dropout is rare. Jeno et al. (2018) present results on 754 biology students in Norway from a cross-sectional study, showing a negatively association from perceived competence and autonomous motivation on dropout intentions. Furthermore, in an indirect effect, relatedness predicts dropout through perceived competence and autonomous motivation. Another recent study is one by Teuber et al. (2021), which is based on a survey with cross-sectional design of 477 participants, of whom 89% were at university, the rest being at college from different disciplines all over Germany. The need for competence is in this research found to be associated with lower scores on intention to drop out. Furthermore, Klein (2019) shows a negative association between integration, seen as relatedness, and dropout intention from 13,315 enrolled students in 33 higher education institutions.

### Basic needs theory and interest

Based on the theoretical assumptions of POI, the development of interest is grounded on two components (Krapp, 2005). The first is a cognitive-rational factor; the second is an emotional subsystem. Both components make an important contribution on the development of interest. The combination of these two factors is seen as a mechanism of system regulation. A part of the emotional subsystem is BPN (Krapp, 2005).

According to Lettau (2018) as well as Renninger and Riley (2013), some research about cognitive-rational factors exist and are seen from the background of motivation and volition theories. However, little research has been done on emotional subsystems, such as BPNs. There exists a view which raises challenges from a theoretical and empirical background. For example, the quality of the experience is biased and difficult to measure, due to the handling of information processes and memories (Lettau, 2018). As consequence, there is less empirical research in this research field.

The few existing studies support the relevance of the emotional aspects of interest. Research with a cross-sectional study done by Keddi (2008) presents associations between BPNs and occupational interest. By measuring data at five timepoints in a project over six months, Minnaert et al. (2011) at least partly vindicated the assumption that the three BPNs influence situational interest. Further longitudinal research is also able to show effects of BPNs on interest. Ferdinand (2014) has demonstrated the impact of autonomy on the longitudinal development of subject interest, by six measurement points in a secondary school study.

### Interest and dropout

A negative relation between interest and dropout, in detail lower interest results in higher dropout, could be explained by the theoretical assumption based on situated expectancy-value theory. Here it is argued that expectancies as well as intrinsic, attainment, utility, and cost values are the determinants of task and activity choice, performance, and engagement in one’s chosen activities (Eccles & Wigfield, 2020). Intrinsic value is seen here, on the one hand, as the expected enjoyment in doing a task for gaining a decision and, on the other hand, the enjoyment someone experiences in doing the task (Eccles & Wigfield, 2020). Interest is assigned to intrinsic values.

Empirical research underlines the relevance of interest to dropout. Schnettler et al. (2020) collected data from students enrolled in mathematics as well as law and questioned them three times during one semester. The result was an intra-individual change from intrinsic value to intra-individual changes in dropout intention that was negative. Research done by Heublein et al. (2017) demonstrates a correlation between decreasing subject interest and dropout, with high relevance for the subjects of economy and social science at traditional universities. Based on analyses of data from the National Educational Panel Study, Behr et al. (2019) infer that the most important reasons for dropout are related to interest, expectations, and aspects of student performance. Powers and Watt (2021) collected data in vocational training every half year over a time period of three years, i.e., six times in total, and found that decreasing interest is associated with dropout intention.

### The present study

The purpose of this study is to understand the factors influencing student dropout. We are interested in determining which factors are relevant to the motivational aspects of decreases in dropout rates. A further aim is to model a process of student dropout based on the motivation theoretical framework, in an explorative way, and to analyze what sustains motivation in the long term. Research has shown that motivation decreases during the first academic year and is associated with dropout intention (Benden & Lauermann, 2021; Grassinger, 2018). In light of this, we focus our research on the first academic year. Our assumptions are that the three BPNs (Ryan & Deci, 2017a) determine subject interest (Eccles & Wigfield, 2020; Krapp, 2005) and finally effect dropout. Furthermore, we test for indirect effects of BPNs on dropout via subject interest. The results of empirical research support these assumptions. In particular, we test the following hypothesis.

- *Hypothesis 1*: higher satisfaction of BPNs, i.e., autonomy, competence, and relatedness, is negatively associated with dropout.
- *Hypothesis 2*: higher satisfaction of BPNs, i.e., autonomy, competence, and relatedness, is positively associated with subject interest.
- *Hypothesis 3*: higher subject interest is negatively associated with dropout.
- *Hypothesis 4*: higher satisfaction of BPNs, i.e., autonomy, competence, and relatedness, is negatively associated with dropout via subject interest.

**Fig. 1:**
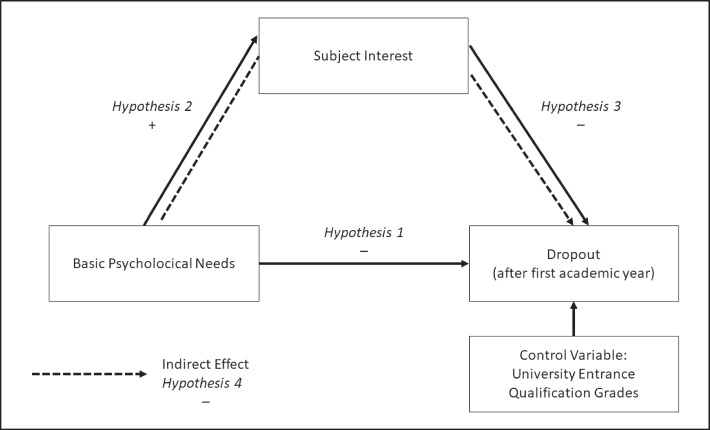
Hypothesized model

We control the grades of the universtiy entrance qualification in our analysis. Current research supports this (Allensworth & Clark, 2020). Furthermore, socioeconomic status (SES) would be a worth control variable due to its effects on motivation (Jansen et al., 2022; Tucker-Drob et al., 2012), achievement (Sirin, 2005; Steinmayr et al., 2012; Tucker-Drob & Harden, 2012), and dropout (Müller & Klein, 2022; Mϋller & Schneider, 2013). However, accurate measures for SES are not available in our data. Figure 1 presents a visualized overview for our four hypotheses.

## Method

### Participants and design

Our data are from the panel study entitled, “Study Process – Crossroads, Determinants of Success and Barriers during a Study at the DHBW” (Deuer & Meyer, 2020). Subjects are students enrolled in their first academic year at the Baden-Wuerttemberg Cooperative State University (DHBW). Data was collected in March 2019 (Wave 4) using an online questionnaire. We matched our survey data at the end of the academic year (September 30th, 2019) with data from the university’s administration. Accordingly, a cross-sectional design is used.

The *N* = 2662 participants in the sample have an average age of *M* = 21.90 years (*SD* = 3.17) with 1285 male (48%) and 1377 female (52%) students. At least 42% of the students have an academic family background, meaning at least one parent has a university degree. The distribution to faculty shows that 58% of the students study business administration, 33% are engineering students, and 9% are enrolled in the academic field of social work.

The educational program for the cooperative students in this sample rotates every three months. It varies between academic learning of theoretical frameworks at university and practical experience in companies. From the background of this special situation and based on a high proportion of practice, this bachelor program awards 210 credits over 36 months, according to the European Credit Transfer System (Wild & Neef, 2019). The DHBW was the first university in Germany to offer a cooperative education program (Huf, 2004) and currently has the most cooperative students (nearly 34,000) in Germany (Winkelmann et al., 2021). Furthermore, the cooperative education system of DHBW has been transferred to numerous countries, like Thailand and China (Reinhard & Gerloff, 2020). The number of cooperative students in Germany has more than doubled over a period of the last 10 years up to 108,202 in the year 2019 (Federal Institute for Vocational Education and Training, 2021).

### Measures

We used McDonald’s omega to estimate reliability in our sample (McDonald, 1999), with a value of ω ≥ 0.70 considered acceptable (Viladrich et al., 2017). These scales for BPNs and subject interest use items with a 5-point Likert scale ranging from 1 (= strongly disagree) to 5 (= strongly agree).

#### Basic psychological needs

We measure the three basic psychological needs using an instrument adapted from Kunter (2005). For competence, reliability is seen as problematic (ω = 0.69; four items; item sample: In the theory phase, I am trusted with difficult tasks). The measure of autonomy is seen as acceptable (ω = 0.77, four items; item sample: In the theory phase, we are encouraged by the lecturers to find our own solutions). Reliability for relatedness is seen as excellent (ω = 0.90; four items; item sample: In the theory phase, I feel I belong to the class).

#### Subject interest

In this study, subject interest is measured by an instrument adapted from Fellenberg and Hannover (2006). Reliability in our sample is seen as good (ω = 0.84; sample item: My field of study is just right for me). The scale has four items.

#### Dropout

In the line with Larsen et al., (2013, p. 5), the “term ‘university dropout’ is commonly used to describe situations where a student leaves the university study in which (s)he has enrolled before having obtained a formal degree.” In our study, we follow this definition. We use data with dropout information from university administration at the end of the first academic year. The dichotomous values for this variable in our data are zero (= no dropout) and one (= dropout). In total, our dataset includes 161 persons who dropped out of university.

#### University entrance qualification grades

The measures of university entrance qualification grades are obtained through the university administration. In the German education system, the university entrance qualification grades vary between the best performance score of 1 (equivalent to a grade of A in Great Britain and in the US) and the lowest performance score of 4 (equivalent to an E in Great Britain and a D in the US). We recode this measurement in our analysis, so that higher scores indicate better performance.

#### Measurement invariance testing of basic psychological needs and subject interest for dropout

We perform analyses of measurement invariance for basic psychological needs and subject interest, in order to assess the (psychometric) equivalence of constructs across dropout and no dropout groups; in addition, we check that a construct has the same meaning in those groups, by following four steps from Putnick and Bornstein (2016):

1. configural, equivalence of model form; (2) metric (weak factorial), equivalence of factor loadings; (3) scalar (strong factorial), equivalence of item intercepts or thresholds; and finally (4) residual (strict or invariant uniqueness), equivalence of items’ residuals or unique variances. (p. 2)

To judge whether differences in model fit were significant, we follow the cut-off values suggested by Chen (2007), interpreting a decline of *CFI* ≥ 0.010 and *RMSEA* ≥ 0.015 or *SRMR* ≥ 0.010 as indicative of noninvariance (for metric invariance: *SRMR* ≥ 0.030). The results confirm strict measurement invariance for persons with dropout, compared with persons with no dropout (see Table 1).

**Table 1:** Results of measurement invariance testing between students` dropout and no dropout (*N* = 2662)

	*df*	χ^2^	χ^2^/*df*	*CFI*	*TLI*	*RMSEA*	*SRMR*	Δ *CFI*	Δ *RMSEA*	Δ *SRMR*
Configural invariance	196	1994.83	10.18	.954	.944	.055	.035			
Metric invariance	208	1005.17	4.83	.954	.947	.054	.035	.000	.001	.003
Scalar invariance	220	1048.91	4.77	.953	.948	.053	.036	.001	.001	.001
Strict invariance	236	1109.68	4.70	.950	.949	.053	.036	.003	.000	.001

*n*(no dropout) = 2501, *n*(dropout) = 161

### Data analyses and missing values

We use SPSS (Version 28) to explore data in the chapter preliminary analysis. Pearson’s *r* is interpreted as per Cohen (1988); values from 0.10 to 0.29 are seen as small, between 0.30 to 0.49 as medium, and ≥ 0.50 as large. Analyzing the effects of the *t*-tests, we use Hedges *g* instead of Cohen’s *d*, due to the adjustment for unequal sample sizes with Hedges *g* (Barton & Peat, 2014). The interpretation of Hedges *g* is same as Cohen’s *d*, with 0.20 to 0.49 representing small effect size, 0.50 to 0.79 as medium effect size, and above 0.80 as large effect size (Cohen, 1988). Skewness values falling outside the range of − 1 to + 1 are seen as problematic, as with a normal distribution (Hair et al., 2014). In our data analysis, we see *p*-values of less than or equal to 0.05 (two-tailed) as statistically significant.

We use structural equation modeling (SEM; Ullman, 2013) and the package “lavaan” by Rosseel (2012) in software R to test our hypothesis. Due to the dichotomous outcomes of the dependent variable dropout, we calculate our estimations by diagonally weighted least squares (*DWLS*). The criteria by Hu and Bentler (1999) with *RMSEA* ≤ 0.06 and *CFI* and *TLI* ≥ 0.95, as well as *SRMR* ≤ 0.08, are seen as constituting a good model fit. Furthermore, we report weighted root mean square residual (*WRMR*), where values < 1.0 have been suggested as indicative of adequate model fit (Yu, 2002). In testing hypotheses 1–3, we use direct effects. Our analysis yields standardized coefficients here. In alignment with Hayes (2018), we tested the mediator effect (hypothesis 4) by estimating bootstrapped conditional indirect effects (using 5000 replications) for this analysis; we use non-standardized effects for a better interpretation. The zero hypothesis of a non-indirect effect is rejected in this approach when the confidence interval does not integrate the value zero.

The use of cross-sectional analysis for longitudinal mediation processes is problematic and highly debated (Fairchild et al., 2017; O’Laughlin et al., 2018). The results of such analyses should be interpreted very cautiously. Since our research claims an exploratory character and results in this research field are rare, we apply this analysis strategy.

The sample of 2662 participants has missing values, in the range for the variables between 0 and 16% (*M* = 9.00; *SD* = 4.20). For 2097 participants (79% of the sample), no missing values exist. We replaced the missing data using multiple imputation by chained equations of the R package “mice,” with 20 imputations (van Buuren & Groothuis-Oudshoorn, 2011).

## Results

### Preliminary analysis

Table 2 presents descriptive information and correlations (*r*) of the continuous variables in our study. There exists a large effect between autonomy and competence (*r* = 0.58). Autonomy shows a medium effects size with relatedness (*r* = 0.30) as well as with subject interest (*r* = 0.34). Competence and relatedness show a medium effects size (*r* = 0.35) as well. Relatedness has a left-skewed distribution (skew =  − 1.35) and kurtosis = 2.34, which is seen as problematic, given the assumption of a normal distribution.

**Table 2:** Descriptive statistics and bivariate correlations (*r*)

	*M*	*SD*	*Skew*	*Kurtosis*	1		2		3		4	
1. Autonomy	3.36	0.80	− 0.35	0.01	−							
2. Competence	3.85	0.67	− 0.63	0.86	.58	***	−					
3. Relatedness	4.22	0.77	− 1.35	2.34	.30	***	.35	***	−			
4. Subject interest	3.84	0.71	− 0.74	0.93	.34	***	.29	***	.26	***	−	
5. UEQG	2.79	0.61	− 0.10	− 0.71	− .11	***	− .04		− .01		− .04	*

*UEQG*, university entrance qualification grades. *N* = 2662. Scales ranging from 1 (= strongly disagree) to 5 (= strongly agree). UEQG ranging from 1 (= lowest performance) to 4 (= best performance); *p < .05; **p < .01; ***p < .001

**Fig. 2:**
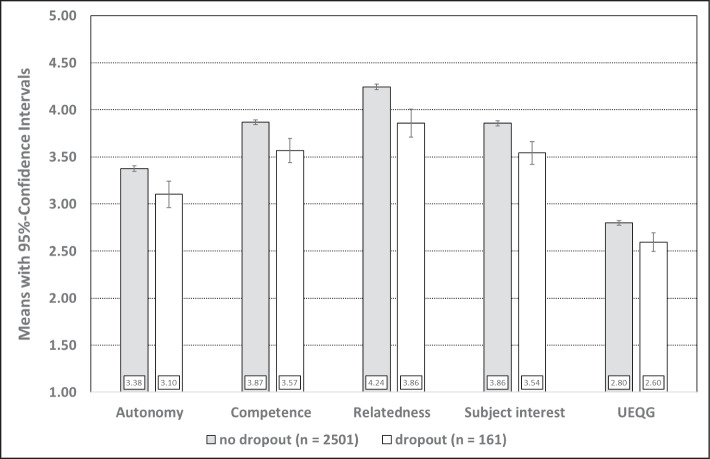
Differences between dropout compared with no dropout in basic need satisfaction, subject interest, and university entrance qualification grades (UEQG) for *N* = 2662 participants. Scales ranging from 1 (= strongly disagree) to 5 (= strongly agree). UEQG ranging from 1 (= lowest performance) to 4 (= best performance)

For no dropout compared to dropout, we identify mean level differences (see Fig. 2). Persons with no dropout always show higher scores than persons with dropout, with significant small effect size. In particular, this can be seen for autonomy (*t*(2660) = 4.23, *p* ≤ 0.001, Hedges *g* = 0.34), competence (*t*(2660) = 5.54, *p* ≤ 0.001, Hedges *g* = 0.45), relatedness (*t*(2660) = 6.17, *p* ≤ 0.001, Hedges *g* = 0.50), subject interest (*t*(2660) = 5.47, *p* ≤ 0.001, Hedges *g* = 0.44), and university entrance qualification grades (*t*(2660) = 4.11, *p* ≤ 0.001, Hedges *g* = 0.33).

### Results on the hypothesis

**Fig. 3:**
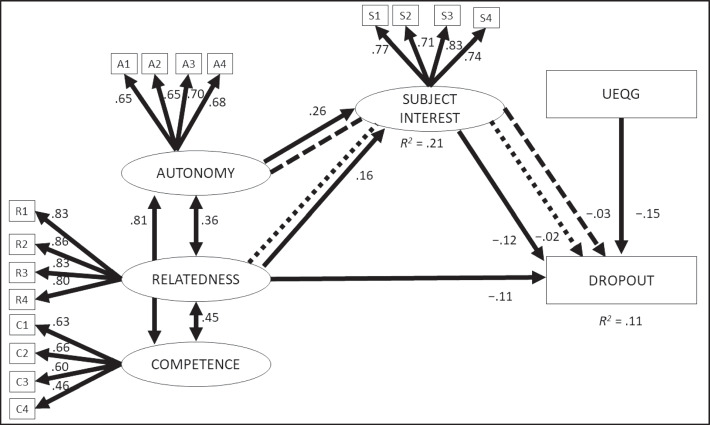
Model for the direct and indirect effect of basic psychological needs on dropout through subject interest (*N* = 2662). Solid lines are direct effects significant at the significance level of *p* ≤ .05. The two indirect effects at the significance level of *p* ≤ .05 are presented with dotted and dashed lines. Coefficients are standardized beta weights. UEQG are university entrance qualification grades

We use the structural equation modeling technique to test our hypothesis. Fit indices of the estimation show adequate fit (χ^2^ = 537.231; *df* = 126; χ^2^/*df* = 4.264; *p* ≤ 0.001; *CFI* = 0.985; *TLI* = 0.984; *RMSEA* = 0.035; *SRMR* = 0.034; *WRMR* = 1.700). This result indicates that the theoretical model is accurately and reliably representing the data (see Fig. 3).

Our results show that only relatedness has a significant association with dropout (β =  − 0.11; *p* = 0.023). Autonomy (β = 0.03; *p* = 0.787) and competence (β =  − 0.18; *p* = 0.120) have no significant association with dropout. Autonomy (β = 0.26; *p* ≤ 0.001) and relatedness (β = 0.16; *p* ≤ 0.001) are associated with subject interest, whereas competence has no significant association with subject interest (β = 0.12; *p* = 0.071). There is also a negative significant association between subject interest and dropout (β =  − 0.12; *p* = 0.006). Our results show a high covariance between autonomy and competence (*r* = 0.81; *p* ≤ 0.001) in the model.

Analysis of the 95%-confidence intervals for the indirect effects confirmed mediation. Subject interest mediates the effect of autonomy on dropout (*b* =  − 0.05, 95% CI = [− 0.09; − 0.01]). Furthermore, there exists an indirect association from relatedness on dropout via subject interest (*b* =  − 0.03, 95% CI = [− 0.05; − 0.01]).

## Discussion

The aim of this paper is to identify the motivational predictors of dropout. We examine the entry phase of a study program, because current research has shown that motivation decrease fast in this phase and this is associated with dropout (Benden & Lauermann, 2021; Grassinger, 2018). Here, we were primarily interested in exploring possible motivational processes relating to dropout. We did this by testing the research question: How do BPNs and subject interest affect dropout?

Our findings highlight the relevance of motivational elements relevant to dropout. Hypothesis 1 is confirmed for the negative association between relatedness and dropout. We can confirm hypothesis 2, regarding an association between autonomy and relatedness with subject interest. In addition, we can confirm hypothesis 3 that subject interest is associated with dropout. Furthermore, we are able to confirm hypothesis 4, that there is an indirect effect from autonomy and relatedness on dropout via subject interest.

The present results should be seen in the context of the theoretical assumptions on the subject. The analyzed associations of BPN to dropout underline the importance of the BPN assumption in the six mini-theories of SDT (Ryan & Deci, 2017a; Sheldon & Prentice, 2019). The results once again confirm that satisfying BPN have a positive association to performance (Ryan & Deci, 2017b). The detailed results are partially in line with former research. For example, Klein (2019) found a similar effect from relatedness as well as integration on dropout. Furthermore, Resch et al. (2022) present results along the same lines. However, research done by Jeno et al., (2018, 2021) or Lavigne et al. (2007) show a further association from competence and autonomy on dropout intention as well as academic success that is not seen in our research. However, it can be said that the satisfaction of BPN should be seen as important component of academic success. Maybe, a carefully trend that relatedness is potentially the most important factor on dropout when analyzing BPN on dropout could be identified.

Our research contributes to explain development of motivation in an academic context. According to Krapp (2005) and POI, we can underline the relevance of emotional component, here BPN, beside cognitive-rational factor. We found in our research direct associations between autonomy and subject interest as well as between relatedness and subject interest, confirming previous research (c.f. Ferdinand, 2014; Minnaert et al., 2011). Other research shows that all three BPN influence intrinsic motivation, where interest can be seen as a component of intrinsic motivation (Karimi & Sotoodeh, 2019). Same study present results, that BPN influence academic engagement indirectly through intrinsic motivation (Karimi & Sotoodeh, 2019).

In line with expectancy-value theory, we found that subject interest, seen as intrinsic value, is associated with dropout. Thus, we were able to integrate one of four value variables from this theoretical model into our analysis. Consequently, we were able to demonstrate the adequate usefulness of this theoretical model in explaining academic success in the context of achievement motivation. Our research is supported by the longitudinal results of Schnettler et al. (2020), as well as Benden and Lauermann (2021), who were also able to demonstrate the significance of the intrinsic values on the dropout intention.

Several practical implications can be derived from our research. One important recommendation here involves strengthening the three BPNs while supporting and supervising students. We believe this can be done in various ways. One way to do this is to improve lecturers’ quality of instruction, e.g., by offering didactic training to raise teachers’ awareness of the BPNs. Quality of instruction is very important, because it is also associated with dropout (Blüthmann et al., 2011; Georg, 2009). Another partial remedy involves supporting students in their academic and social integration. There exist several evaluated approaches and arrangements, such as remediation (Bettinger & Long, 2009; Tieben, 2019), attending to the composition of groups in teaching courses (Booij et al., 2017), student-faculty mentoring (Sneyers & de Witte, 2018), and academic advising (Kot, 2014). A third way to keep the motivation of students high throughout the entirety of their study programs are motivation programs, with coaching and self-assessment of students’ strengths and weaknesses (Bettinger & Baker, 2014; Ćukušić et al., 2014; Unterbrink et al., 2012).

The strength of this study is that it combines survey data and data from university administration. So, measurement error and social desirability is reduced. For example, most studies, such as those of Dresel and Grassinger (2013) and Schnettler et al. (2020), only measure dropout intention, and Deuer and Wild (2019) demonstrate the low prognostic validity of dropout intention for assessing real dropout. The quality of our psychometric instruments in our sample for the instruments used for BPNs and subject interest is seen as decent. We can assume strict measurement invariance between dropout and no dropout for the psychometric measures. Furthermore, the theoretical assumptions are acquitted well by the empirical data, as is indicated in the measures of fit in the structural equation model. Our sample is not biased by the Covid 19 pandemic, as we performed our survey in 2019, prior to the pandemic. We test the theoretical framework in subpopulations, cooperative students, that are getting more important in the next year, as more and more students enroll in this study program in Germany (Federal Institute for Vocational Education and Training, 2021).

Our research has limitations. We are using data from only one university with 12 campuses in one federal state of Germany. Furthermore, the generalizability from our sample to all students is problematic, because we collect data from cooperative students who are chosen from companies; as a consequence, a possible selection bias exists (Kupfer, 2013; Wild & Neef, 2019). Thus, our research has the character of a case study. Another problem is seen in the fact that we only could integrate 161 persons with dropout in our analysis; thus, the meaningfulness of our analyses is low for this subgroup. Moreover, King and Zeng (2001) see few events in such binary data as problematic for estimating statistical models. However, they emphasize an existing fear of this problem and the consequence that data with so few events in such situations are rarely measured. With our research, we try to counteract this situation. Moreover, our research is based on a cross-sectional design, and longitudinal research is needed for exact, informative modeling processes. The high correlation between autonomy and relatedness (*r* > 0.80) in our SEM is seen as problematic, because of indicating measuring similar constructs, multicollinearity and consequently endangered inference process (Hair et al., 2014). Finally, the reliability of measuring the BPN competence, with ω = 0.69 in our sample, is also seen as problematic, because it is low and may consequently be not significantly associated with subject interest (β = 0.12; *p* = 0.071) or dropout (β =  − 0.18; *p* = 0.120). Here, it seems possible, on the one hand, that the experience of competence actually has a small influence on subject interest and dropout risk, which would require further theoretical explanation; on the other hand, it also seems possible that a better operationalization of the construct is needed to uncover the interrelationships. Therefore, future research should analyze the effect of competence on subject interest as well as dropout in greater detail.

New research questions arise from our findings. One new research interest is if differential effect sizes for the motivational process of dropout exist, such as gender or domains (Jansen et al., 2016; van Maurice et al., 2014). From the theoretical background of situated expectancy-value theory, it must be emphasized that not only intrinsic value should be integrated into research, but also attainment value, utility value, and costs; they, too, should be analyzed for a mediation variable in dropout processes between BPNs and dropout (Eccles & Wigfield, 2020). However, the observed low variance in the variable dropout of nearly 11% in our structural equal model indicates that there are further variables which affect dropout, e.g., financial aspects, which need to be investigated (Kehm et al., 2019; Neugebauer et al., 2019; Seidman, 2012). All things considered, dropout seems to be affected by different factors (Heublein, 2014), and our work is only one small step toward a better understanding of their relevance and interactions to student dropout. Working on the research question of what sustains motivation in university in the long-term is necessary to minimize dropout.

## Conclusion

Our work advances research on the role of motivation in student dropout. We found that the satisfaction of BPNs is associated with dropout via subject interest. Arrangements to prevent dropout, such as increasing relatedness through mentoring programs, are discussed here. Further research is needed, as there are various other factors explaining dropout processes.

## Data Availability

Data is available. Please contact the corresponding author.

## Code availability

Syntax is available. Please contact the corresponding author.
